# Supplementary material for: Accuracy of Thoracic Ultrasonography for the Diagnosis of Pediatric Pneumonia: A Systematic Review and Meta-Analysis
Source: Diagnostics (Basel). 2023 Nov 16;13(22):3457. doi: 10.3390/diagnostics13223457 (PMC10670251; doi:10.3390/diagnostics13223457)
Supplement: Supplementary file 1 [file diagnostics-13-03457-s001.zip › diagnostics-2667447-supplementary.pdf]

Search strategies：

PubMed:

#1"Adolescent"[Mesh] OR "Child"[Mesh] OR "Infant"[Mesh] OR Children[Title/Abstract] childhood[Title/Abstract] OR child[Title/Abstract] "pediatric age"[Title/Abstract] OR "pediatric patient*"[Title/Abstract] OR "pediatric age group"[Title/Abstract] OR "febrile children"[Title/Abstract] OR "hospitalized children"[Title/Abstract] OR "hospitalised children"[Title/Abstract] OR infant*[Title/Abstract] OR neonatus[Title/Abstract] OR neonate[Title/Abstract] OR adolescent*[Title/Abstract] OR Teenager*[Title/Abstract] OR Teen*[Title/Abstract] OR youth*[Title/Abstract] OR "pediatric ward"[Title/Abstract] OR "pediatric emergency department"[Title/Abstract]

#2"Pneumonia"[Mesh] OR Pneumonia[Title/Abstract] OR "community-acquired pneumonia"[Title/Abstract] OR "community acquired pneumonia"[Title/Abstract] OR CAP[Title/Abstract] OR "paediatric pneumonia"[Title/Abstract] OR "neonatal pneumonia"[Title/Abstract] OR "suspected pneumonia"[Title/Abstract] OR "infectious pneumonia"[Title/Abstract]

#3"Ultrasonography"[Mesh] OR Ultrasonography[Title/Abstract] OR Ultrasound[Title/Abstract] OR sonography[Title/Abstract] OR "lung ultrasonography"[Title/Abstract] OR "lung ultrasound"[Title/Abstract] OR "lung sonography"[Title/Abstract] OR LUS[Title/Abstract] OR "Pulmonary ultrasound"[Title/Abstract] OR "Pulmonary ultrasonography"[Title/Abstract] OR "thoracic ultrasonography"[Title/Abstract] OR "thoracic ultrasound"[Title/Abstract] OR TUS[Title/Abstract] OR "chest ultrasonography"[Title/Abstract] OR "chest ultrasound"[Title/Abstract] OR "diagnostic ultraso*"[Title/Abstract] OR "Point-of-care ultraso*"[Title/Abstract] OR "sonographic finding"[Title/Abstract]
#1 AND #2 AND #3

Embase:

#1 'Child'/exp OR 'Child':ab,ti OR 'Children':ab,ti OR 'childhood':ab,ti OR 'Infant':ab,ti OR 'Adolescent':ab,ti OR 'hospitalized children':ab,ti OR 'hospitalized child':ab,ti OR 'hospitalised children':ab,ti OR 'Teenager':ab,ti OR 'Teen':ab,ti OR 'youth':ab,ti OR 'pediatric patient':ab,ti OR 'pediatric age group':ab,ti OR 'febrile children':ab,ti OR 'febrile child':ab,ti OR 'neonatus':ab,ti OR 'neonate':ab,ti OR 'pediatric emergency department':ab,ti OR 'pediatric ward':ab,ti

#2 'Pneumonia'/exp OR 'Pneumonia':ab,ti OR 'community-acquired pneumonia':ab,ti OR 'community acquired pneumonia':ab,ti OR 'CAP':ab,ti OR 'paediatric pneumonia':ab,ti OR

'neonatal pneumonia':ab,ti OR 'suspected pneumonia':ab,ti OR 'infectious pneumonia':ab,ti

#3 'Ultrasonography':ab,ti OR 'Ultrasound':ab,ti OR 'sonography':ab,ti OR 'lung ultrasonography':ab,ti OR 'lung ultrasound':ab,ti OR 'lung sonography':ab,ti OR 'LUS':ab,ti OR 'Pulmonary ultrasound':ab,ti OR 'Pulmonary ultrasonography':ab,ti OR 'thoracic ultrasonography':ab,ti OR 'thoracic ultrasound':ab,ti OR 'TUS':ab,ti OR 'chest ultrasonography':ab,ti OR 'chest ultrasound':ab,ti OR 'diagnostic ultrasound':ab,ti OR 'diagnostic ultrasonography':ab,ti OR 'Point-of-care ultrasound':ab,ti OR 'Point-of-care ultrasonography':ab,ti OR 'sonographic finding':ab,ti
#1 AND #2 AND #3

Web of science:

#1 TI=(Child) OR TI=(Children) OR TI=(childhood) OR TI=(Infant) OR TI=(Adolescent) OR TI=(hospitalized children) OR TI=(hospitalized child) OR TI=(hospitalised children) OR TI=(Teenager) OR TI=(Teen) OR TI=(youth) OR TI=(pediatric patient) OR TI=(pediatric age group) OR TI=(febrile children) OR TI=(febrile child) OR TI=(neonatus) OR TI=(neonate) OR TI=(pediatric emergency department) OR TI=(pediatric ward)

#2 TI=(Pneumonia) OR TI=(community-acquired pneumonia) OR TI=(community acquired pneumonia) OR TI=(CAP) OR TI=(paediatric pneumonia) OR TI=(suspected pneumonia) OR TI=(neonatal pneumonia) OR TI=(infectious pneumonia)

#3 TI=(Ultrasonography) OR TI=(Ultrasound) OR TI=(sonography) OR TI=(lung ultrasonography) OR TI=(lung ultrasound) OR TI=(lung sonography) OR TI=(LUS) OR TI=(Pulmonary ultrasound) OR TI=(Pulmonary ultrasonography) OR TI=(thoracic ultrasonography) OR TI=(thoracic ultrasound) OR TI=(TUS) OR TI=(chest ultrasonography) OR TI=(chest ultrasound) OR TI=(diagnostic ultrasound) OR TI=(diagnostic ultrasonography) OR TI=(Point-of-care ultrasound) OR TI=(Point-of-care ultrasonography) OR TI=(sonographic finding)
#1 AND #2 AND #3
